# Climate and health capacity building for health professionals in the Caribbean: A pilot course

**DOI:** 10.3389/fpubh.2023.1077306

**Published:** 2023-01-26

**Authors:** Cecilia Sorensen, Nicola Hamacher, Haley Campbell, Paula Henry, Keriann Peart, Loren De Freitas, James Hospedales

**Affiliations:** ^1^Global Consortium on Climate and Health Education, Columbia University, New York, NY, United States; ^2^Department of Emergency Medicine, Columbia Irving Medical Center, New York, NY, United States; ^3^Department of Environmental Health Sciences, Mailman School of Public Health, Columbia University, New York, NY, United States; ^4^Mailman School of Public Health, Columbia University, New York, NY, United States; ^5^EarthMedic/Nurse Foundation for Planetary Health, Port of Spain, Trinidad and Tobago

**Keywords:** climate and health education, health profession courses, Caribbean, vulnerability, evaluation

## Abstract

Climate change is a reality in the Caribbean and its effects are already harming health, yet the health workforce capacity to implement climate mitigation and adaptation measures is lacking. From March-May of 2022, a free, live-virtual, evidence and competency based 10-week climate and health course targeted toward health risks in the Caribbean was deployed to: (1) increase communication about climate and health, (2) equip health professionals with knowledge and skills that could be readily incorporated into practice, and (3) engage health professionals with climate and health initiatives within their communities. Participants in this course came from 37 countries, 10 different health-related fields, and five different general places of work. Longitudinal surveys revealed significant changes in health professional communication, engagement and application of climate and health knowledge and skills. Live-virtual, evidence and competency-based courses, regional-specific courses have the potential to change health professional behaviors toward addressing climate impacts on health.

## Introduction

Climate change is a health issue that affects morbidity, mortality, and society's abilities to deliver healthcare and support healthy living. The effects of climate change are already harming health in the Caribbean region and impacts will only intensify in the coming years (1). Within the region, heat waves, hurricanes and rainstorms are becoming more deadly (2), disease outbreaks last longer and are seen in new regions (3–6), wildfire smoke and Saharan dust reduces air quality (7, 8), and food and water security are threatened by extreme weather and floods (9). The Caribbean community of nations, all classified as Small Island Developing States (SIDS), will face escalating risks with the changing climate, placing the provision of health care and the prevention of communicable and non-communicable diseases in jeopardy. The Caribbean SIDS include low-lying coastal countries like Guyana, Suriname, and Belize which share similar sustainable development challenges, including small populations, limited resources, and susceptibility to climate change.

The ability to preserve and protect health in the Caribbean is further complicated by insufficient research into the substantive health impacts from climate change in the region and a lack of evidence-based actions to prevent and prepare against these health impacts (10). Furthermore, while climate change adversely impacts health in many ways, the health system globally contributes an estimated 4.4% of global carbon emissions, with energy generation and distribution being the largest contributor. Switching to renewables such as solar power in health facilities would help reduce operational costs, cut carbon emissions, and ensure continuity of critical patient care services in the event of extreme weather and blackouts. There is an imperative for quick action on many fronts: to prevent climate change at its source by reducing heat-trapping greenhouse gas emissions; to increase the research base regarding the impacts of climate change on the health of Caribbean populations, to recognize, prevent and respond to climate-health impacts at individual and population levels; to build climate resilient and low carbon health systems and to communicate effectively about these issues for the sake of safeguarding human health.

Health professionals occupy a critical position in the response to climate change. First, they are charged with protecting individual and community health in the face of multiple new and compounding health risks that will become more costly and complicated to address as time goes on. For example, Public Health professionals will be tasked with creating vulnerability assessments to and performing impacts assessments resulting from climate-related events. Clinical health professionals will increasingly care for patients whose disease processes are caused or accelerated by climate change and will be tasked with counseling and treating these individuals as well as readying healthcare systems to cope with increasing burdens of disease (11).

Second, health professional expertise must be brought to bear on cross-sectoral solutions to the climate crisis, and to articulate climate risks and solutions to patients, the public and policy makers. Third, the institutional knowledge of health professionals is indispensable in modifying health systems and communities to become both resilient to climate threats and environmentally sustainable (12). However, as climate change is currently outside of the traditional training and continual professional development of the health workforce, few have the skills and expertise to effectively fill these critical new health professional roles that are essential to protect health in the face of the climate crisis.

Several Caribbean ministries of health, in partnership with the World Health Organization, have performed national climate vulnerability assessments and identified training health professionals, as a means to strengthen health systems, a priority (13–16). Furthermore, researchers, students, and other professionals have identified a need for improved climate and health education in professional training. (17). However, there have been limited efforts to standardize content or ensure that educational efforts change practical actions, especially in the Caribbean region. To address this gap, The Caribbean Climate and Health Responder Course was developed through a partnership between EarthMedic and EarthNurse (EM&EN), the Caribbean Institute for Meteorology and Hydrology and the Global Consortium on Climate and Health Education (GCCHE), with an aim to increase climate and health knowledge, skills, communication, and action that reflect the crucial emerging roles of health professionals within the Caribbean.

Specifically, the goal of this course was to: (1) Increase health professional communication about climate and health, (2) equip health professionals with knowledge and skills that could be readily incorporated into practice, and (3) engage health professionals with climate and health initiatives within their communities. To evaluate the effectiveness of the course in meeting the stated goals, we conducted a longitudinal study of course participants, to evaluate whether the 15-h live-virtual course has the potential to increase health professional capacity to address climate and health threats.

## Methods

### Program structure

The curricular foundation of this educational initiative was the GCCHE core competencies for health professionals (18), a set of highly-vetted global educational standards which cover climate and health analytic skills and knowledge, communication and collaboration, policy, and public health and clinical practice competencies (17). These competencies were divided into 10 discrete modules (see Appendix 1), each with predetermined learning objectives, which were adapted from the GCCHE competencies by regional experts to meet specific climate and health risks in the Caribbean. The course consisted of two parts:

1) Weekly 60-min core didactic lectures followed by 30 min of moderated question and answer.
2) Bi-monthly, interactive, 90-min “skills and practice” sessions.

All lectures were delivered by local and regional climate and health experts and had accompanying online reading and learning resources. Prior to the course, the expert faculty received a 1-h orientation to the curriculum and the expectations of the course participants. In addition to the 10 core didactic sessions, participants had the option to participate in bi-monthly “skills and practice” sessions, structured around clinical cases, climate tools, communication and leadership strategies, and teaching tools (Appendix 1). On enrollment, all participants received the course syllabus, a resource bank and recommended readings for each session and lecture slides, to jumpstart their climate and health practice. Participants who successfully completed 70% of the didactic training sessions (at least seven sessions) and passed a short test at the end of the 10-week training (with a score of 70% or greater), received a *Climate and Health Responder Certificate of Participation*. Continuing Medical Education Credit was provided by the American Association of Continuing Education (AACME) through the Trinidad and Tobago Medical Association.

### Intended audience

This educational initiative aimed to engage physicians, nurses, allied health professionals, national or local public health workers, hospital administrators, health system leaders, health educators, policymakers, environmental health professionals and government officials. Members of these groups were invited to participate through outreach *via* email and social media to both individuals and groups representing these professions. Outreach and promotion were also done through a promotional video sent to direct contacts with presidents of medical and nursing associations, Chief Medical officers of nation-states within the Caribbean community, regional academic universities, and Pan American Health Organization offices. Regional and global outreach was done through organizations with a focus on health, including the Caribbean Public Health Agency (CARPHA), Healthy Caribbean Coalition (HCC), Caribbean Community Climate Change Center (5Cs) and the Global Climate and Health Alliance (GCHA).

### Recruitment and enrollment

All participants who registered for the course were invited to enroll in the longitudinal study. A pre-course survey was sent *via* email on the first day of the course, using the Qualtrics platform, to all course participants using contact information obtained through Zoom registration. Survey participation was entirely voluntary, however, to increase follow-up, reminders were sent *via* Qualtrics to non-responders. On the last day of the course, all registrants who attended at least one session were sent an email through Qualtrics inviting them to participate in a post-course survey. Response data was collected in Qualtrics and was anonymized before being analyzed. Demographic information (country of residence, occupation, place of work) for each participant who enrolled in the study was obtained from registration information. Only those who attended ≥1 class (demonstrated through Zoom attendance) were counted as “Registered.” Study protocol was approved by Columbia University Institutional Review Board (AAAR4912).

### Course participation

Course participation was recorded in Zoom and made available to the study team following each session. Participation was counted regardless of the duration of attendance for each session. Each session was recorded and immediately posted following each session. Individual asynchronous participation or viewing of course was not tracked.

### Survey description

The longitudinal survey was structured to assess the effectiveness of this training course in affecting professional behavior related to climate and health communication, application of climate and health knowledge skills and engagement or action to address climate and health risks to health.

#### Communication

Survey participants were assessed before and after the course to the degree to which they communicated with patients, community members, and colleagues about the risks of climate change to health. Multiple choice options of frequently, sometimes, rarely, and never were presented.

#### Application

Survey participants were assessed before and after the course regarding the degree to which they incorporated climate change and health knowledge and skills in their professional work environment. Multiple choice options of frequently, sometimes, rarely, and never were presented.

#### Engagement

Survey participants were asked before and after the course how confident they felt that they could engage with a climate and health initiative within their community, institution, or professional area of practice. Multiple choice options of very confident, somewhat confident, not very confident, and definitely not confident were presented.

#### Post-survey questionnaire

The post-course questionnaire included further questions asking for participants to reflect on the effectiveness of the course, how the course may change their practice, their ability to lead adaptation and mitigation initiatives in their communities, and their ability to train other professionals on climate change and health impacts among others (see Appendix 2).

### Analysis

All data from registration, course participation, and both pre- and post-surveys were organized and analyzed using R Studio. For each longitudinal survey question, we calculated the percent change in response for each of the predetermined multiple-choice responses. The number of respondents to each possible answer and total number of respondents was used to calculate percent responses to each question in the pre- and post-surveys. Following these calculations, the pre-course survey percentages and participant numbers were subtracted from post-course survey percentages and participant numbers for each question. This was listed as the “Change” in question and represents the impact of the course on participant responses. Only survey participants who completed both surveys were included in the analysis.

## Results

### Demographics and participation

Participants in this course came from 37 countries, 10 different health-related fields, and five different general places of work (Tables 1–3). The majority of the registered participants were from Jamaica, the United States of America, Trinidad and Tobago, Guyana and the Bahamas. The most common professions represented were physicians and environmental professionals, followed by nurses, and public health practitioners. Nearly half of participants worked for a governmental agency.

**Table 1:** Country of residence of course registrants and survey participants.

**Country**	**Registered**	**Survey participants**	**Country**	**Registered**	**Survey participants**
Jamaica	124	10	Peru	3	0
United States	116	19	Dominica	2	0
Trinidad and Tobago	84	18	Australia	1	1
Guyana	72	15	Cayman Islands	1	1
Bahamas	50	12	Nepal	1	1
Antigua and Barbuda	45	9	Argentina	1	0
Barbados	44	8	Bolivia	1	0
Saint Lucia	34	6	Columbia	1	0
Suriname	28	10	Ecuador	1	0
Grenada	20	6	Fiji	1	0
Haiti	20	1	Jordan	1	0
Puerto Rico	13	3	Malaysia	1	0
Belize	12	4	Mexico	1	0
Virgin Islands (British)	8	2	Montserrat	1	0
Canada	7	1	Netherlands	1	0
United Kingdom	6	1	Nigeria	1	0
Saint Kitts and Nevis	4	1	Saint Vincent and the Grenadines	1	0
Spain	4	1	Turks and Caicos Islands	1	0
Philippines	4	0	Virgin Islands (US)	1	0
Brazil	3	0	Not available	44	2

**Table 2:** Reported occupation of course registrants and survey participants.

**Occupation**	**Registered**	**Survey participants**
Medic/physician	147	33
Environmental health	147	18
Other	117	15
Nurse	100	21
Student	85	15
Public health practitioner	84	14
Mental health	42	5
Emergency responder	22	3
Social worker	13	3
Retired	4	3
Pharmacist	3	2

**Table 3:** Location of work of course registrants and survey participants.

**Place of Work**	**Registered**	**Survey participants**
Governmental agency	335	60
Academic	153	21
Hospital	134	24
Private practice	77	16
CBO/NGO	65	11

In total, 1, 276 individuals registered for the course. Of those, 764 attended classes (i.e., were present for at least one session) and were therefore eligible to participate in the pre-class and post-class surveys. The pre-course survey was completed by 461 participants. The post-course survey was completed by 178 participants. Only participants who completed both surveys, of which there were 132, were used in our analysis.

In Table 2, a large number of registrants listed their occupation as “Student” or “Other.” Table 2 seeks to provide more insight into what these individuals may be studying or working in by listing the location of their work alongside their selected occupation.

### Longitudinal survey

*Communication (Q1):* Compared to the beginning of the course, study participants increased the frequency with which they reported *frequently* discussing climate change and health with their patients, community members, and colleagues by ≈ 9% (*n* = 12). At course completion, 81.8% (*n* = 108) of respondents reported climate and health communications either *frequently* (32.6%, *n* = 34) or *sometimes* (49.2%, *n* = 65) (Table 4).

**Table 4:** Longitudinal questions.

	**Pre-course survey**	**Post-course survey**	**Change**
**Q1: How often do you talk to your patients/community members/colleagues about climate change and health?**	**(** ***n*** **) %**	**(** ***n*** **) %**	**(** ***n*** **) %**
Frequently	(31) 23.5%	(43) 32.6%	(+12) +9.1%
Sometimes	(68) 51.5%	(65) 49.2%	(−3) −2.3%
Rarely	(23) 17.4%	(22) 16.7%	(−1) −0.8%
Never	(10) 7.6%	(2) 1.5%	(−8) −6.1%
**Q2: How often do you incorporate climate change and health knowledge and skills in your work?**	**(** ***n*** **) %**	**(** ***n*** **) %**	**(** ***n*** **) %**
Frequently	(26) 19.7%	(34) 25.8%	(+8) +6.1%
Sometimes	(52) 39.4%	(71) 53.8%	(+19) +14.4%
Rarely	(40)30.0%	(23) 17.4%	(−17) −12.9%
Never	(14)10.6%	(3) 2.3%	(−11) −8.3%
No response	N/A	(1) 0.8%	N/A
**Q3: How confident are you that you can engage with a climate and health initiative (e.g. hospital green team, adaptation project, education) in your community/institution/practice?**	**(** ***n*** **) %**	**(** ***n*** **) %**	**(** ***n*** **) %**
Very confident	(31) 23.5%	(48) 36.4%	(+17) +12.9%
Somewhat confident	(60) 45.5%	(68) 51.5%	(+8) +6.1%
Not very confident	(31) 23.5%	(12) 9.1%	(−19) −14.4%
Definitely not confident	(10) 7.6%	(3) 2.3%	(−7) −5.3%
No response	N/A	(1) 0.8%	N/A

*Application (Q2):* Compared to the beginning of the course, study participants reported a 20.5% increase in *frequently* (+6.1%, *n* = 8)or *sometimes* (+14.4%, *n* = 19) incorporating climate change and health knowledge and skills into their work (Table 4).

*Engagement (Q3):* Compared to the beginning of the course, 87.9% (*n* = 116) participants reported being *very confident* or *somewhat confident* in their ability to engage with climate and health initiatives—a 12.9% (*n* = 17) and 6.1% (*n* = 8) increase from the beginning of the course (Table 4).

### End of course survey

At the end of the course, 50.8% (*n* = 67) of participants felt very prepared to have conversations (about climate change and health) with all contacts and (44.7%, *n* = 59) felt prepared in limited scenarios (Table 5). A similar number (93.9%, *n* = 124) felt that knowledge and skills gained through course participation would change their professional practice to “a large degree” (29.6%, *n* = 39) or in “some aspects” (64.4%, *n* = 85).

**Table 5:** Post-course survey questions.

**Q4: Do you think that this course has prepared you to speak with patients/community members about climate change and their health?**	**(*n*) %**
Yes, I feel very prepared to have conversations with all contacts	(67) 50.8%
Yes, I feel prepared but in limited scenarios	(59) 44.7%
No, I do not feel like I have the expertise to speak with others on this subject	(5) 3.8%
No response	(1) 0.8%
**Q5: Do you think that the knowledge and skills you gained from the Climate and Health Responder Course will change your professional practice?**	**(** ***n*** **) %**
Yes, it will change my practice to a large degree	(39) 29.6%
Yes, it will change some aspects of my practice	(85) 64.4%
Not sure if it will change my practice	(5) 3.8%
My practice will not change	(1) 0.8%
No response	(2) 1.5%
**Q6: Do you think the knowledge that you gained from the Climate and Health Responder Course has prepared you to lead climate and health ADAPTATION initiatives within your community of practice?**	**(** ***n*** **) %**
Yes, I now feel confident leading initiatives	(34) 25.8%
I feel more confident, but still feel like I need more knowledge/experience to serve as a leader	(76) 57.6%
I do not feel confident to serve as a leader, but am more prepared to help in initiatives	(21) 15.9%
I do not feel confident to lead or help develop initiatives	(0) 0%
No response	(1) 0.8%
**Q7: Do you think the knowledge that you gained from the Climate and Health Responder Course has prepared you to lead climate MITIGATION initiatives within your community of practice?**	**(** ***n*** **) %**
Yes, I now feel confident leading initiatives	(30) 22.7%
I feel more confident, but still feel like I need more knowledge/experience to serve as a leader	(80) 60.6%
I do not feel confident to serve as a leader, but am more prepared to help in initiatives	(20) 15.2%
I do not feel confident to lead or help develop initiatives	(1) 0.8%
No response	(1) 0.8%
**Q8: How confident are you now, as compared to before you took the Climate and Health Responder course, that you can train others in at least some aspects of climate change?**	**(** ***n*** **) %**
My confidence has increased a great deal	(67) 50.8%
My confidence has increased slightly	(56) 42.4%
My confidence has not changed	(8) 6.1%
My confidence has decreased slightly	(0) 0%
My confidence has decreased a great deal	(0) 0%
No response	(1) 0.8%

Participants were also asked about their ability to lead both adaptation and mitigation initiatives within their community of practice. Approximately a quarter of participants stated they “now feel confident leading [adaptation] initiatives” (25.8%, *n* = 34). A further 57.6% (*n* = 76) of participants stated that they feel more confident, but still need more knowledge and experience to serve as a leader. Responses to questions about leading mitigation initiatives were relatively similar with 22.7% (*n* = 30) stating they “now feel confident leading [mitigation] initiatives” and an additional 60.6% stating they feel more confident, but still need more knowledge and experience to serve as a leader in mitigation initiatives.

Post-course survey participants were also asked to reflect on how their confidence in training others “in at least some aspects of climate change” had changed since the initiation of the course. Respondents overwhelmingly (93.2%, *n* = 123) felt that the course had increased their confidence to some extent with 50.8% (*n* = 67) stating their “confidence has increased a great deal” and 42.4% (*n* = 56) stating their “confidence has increased slightly”.

## Discussion

Health professionals stand on the front lines of the climate crisis, yet many barriers prevent health professional engagement and meaningful action to mitigate the root causes of climate change and adapt their health practice to protect patients and communities, especially in vulnerable areas. Rapid knowledge dissemination, capacity building and health professional action is needed to protect patients, communities, and health systems. In this course, participants from at least 21 countries, 10 different health-related fields, and a variety of health professional settings reported significant changes in intention to communicate, apply, and engage in climate and health discussions and activities after participating in a 10-week live-virtual course. To our knowledge this is the first study that evaluates the potential for virtual learning opportunities to engage health professionals from diverse backgrounds to respond to the climate crisis and informs future initiatives to build a global climate-ready global health workforce.

In a recent multinational study of health professional's views on climate change and health, although an overwhelming majority perceived a growing concern of health harm on their patients and in their communities, many identified barriers to action, communication, and adaptation of practice (19). Forty-one percent reported a lack of knowledge, 31% believed that engaging with the public would not make a difference, and 22% perceived little support from their peers (19). In this course, we were possibly able to overcome several of these stated obstacles, thus enabling a shift in reported engagement. Firstly, the meeting style setting of the Zoom platform allowed active participation during didactic sessions, through activation of the Chat function and the Q&A sessions, building cohesion and peer support. Secondly, skills and practice sessions were framed around specific proven actions that could be taken by health professionals to affect change locally and globally. Effective communication skills, commonly encountered case-histories and interactive resources provided the forum for overcoming inertia toward the notion that action would not make a difference. Thirdly, this course was based on a rigorous, evidence-based curriculum, yet participants were provided with ample time for questions and discussion clarifying and expanding on lessons learnt. Lastly the course considered the time-constraint of busy professionals. This barrier was highlighted by Kotcher et al. as the greatest hurdle. On-line slide decks and recorded videos of live sessions were stored as a repository for further use. Although culturally appropriate and regionally specific knowledge was not assessed in the Kocher study, we believe the availability of such materials may be a barrier. In this program, the majority of expert lecturers for this course, and the course design, was conceived of by professionals actively working in the Caribbean region. We believe that this contributed to the applicability of the knowledge and skills disseminated in the course.

The course had two other dimensions which will likely aid a stronger response. First, it was a multi-disciplinary audience, so the discussion and chats allowed cross-fertilization between professionals. Second, people with an interest in climate and health from the same country got to know each other and developed relationships which will likely enhance post course collaboration.

### Strengths and limitations

The strengths of this course included: widespread engagement of health professionals from diverse backgrounds and practice environments throughout the Caribbean; strong support from regional health, climate and environmental organizations; deep engagement of experts from within the region who served as course faculty; opportunities for bi-directional engagement among course faculty and participants; and an evidence-based approach which adhered to global competencies for health professionals in responding to the climate and health crisis.

An important limitation to recognize is the self-selection of participants into the course and into the study cohort. Though the course was widely promoted and open to all health professionals, students, or interested individuals, it was primarily advertised through pre-existing organizations focused on climate and which may have inadvertently selected individuals already engaged with or particularly willing to engage in climate change and health related initiatives and education. Additionally, data collection required participants engaging in two optional surveys outside of the course which may have limited respondents to those who were particularly engaged with course material. The online Zoom format of the course also selected for individuals who had consistent internet access and were available during the time the courses were offered. In countries lacking consistent internet service or where Zoom is not enabled—potential participants were unable to engage.

### Next steps and future directions

With the momentum stimulated from this course, in July of 2022, EM&EN invited course participants to take part in regionally based health professional working group to develop ideas, tools and culturally appropriate products on climate and health for practitioners, patients and communities. More than 60 participants responded and have since been active in the production of practitioner, patient and community education and capacity/literacy items, to increase knowledge and activate action plans for further research and application of services addressing climate change and health. Furthermore, the working groups are contributing to a newly launching monthly webinar series on climate change and non-communicable diseases in the Caribbean, which will launch October 27, 2022 through a partnership between EM&EN, the Global Consortium on Climate and Health Education and the Healthy Caribbean Coalition. Furthermore, we plan to repeat this survey among course participants in 6 and 12 months to assess the effectiveness of intervention to result in lasting health professional behavioral changes.

To mount a health system-wide effective response commensurate with the risks posed by climate change, which threatens to disrupt stable and secure livelihoods for all (10), health professions will need to assume new roles and responsibilities. The structure of this course was introductory in nature and geared toward reaching a broad range of health professionals. While this course facilitated an interdisciplinary approach to tackle climate and health challenges, more specific training is warranted for each health profession to continue to improve capacity. Further training is also needed to strengthen partnership skills to help “join up silos” between clinical professionals and environmental professionals, as the former sees the health impacts presenting, while the latter are in a better position to influence the environment, water quality, vector breeding, heat adaptation measures, etc.

Specifically regarding the role of health professionals, there are several opportunities for immediate action (20). First, academic institutions which train health professionals can integrate evidence-based climate and health knowledge and skills into the explicit training of all trainees. Such educational training can and should be supported by governing bodies which accredit health professional training institutions as well as provide licensing for individual professionals. Second, a parallel effort is needed to train clinical health professionals already in practice. This might be achieved through partnerships with local and regional health ministries as well as health professional societies who already routinely provide education and training to the health professional workforce. These education efforts can be incorporated into required ongoing maintenance of certification efforts. Importantly, efforts to build capacity within national health sectors to address the climate crisis are routinely incorporated into Health National Adaption Plans (H-NAPS), a subset of Nationally Determined Contributions for the United Nations Framework Convention on Climate Change, and thus might benefit from national level support.

With the success of this program, similar efforts are being initiated in other global regions through the Global Consortium on Climate and Planetary Health Education with local partners in Southeast Asia and Sub-Saharan Africa. Exceptional collaboration, knowledge sharing, and workforce capacity building are essential to tackle the complex ways in which climate change threatens health.

## Conclusion

Climate change is a reality in the Caribbean that will continue to have a significant impact on human health and the health sector. Health professionals occupy a critical position in the response to climate change, including climate mitigation and adaptation, and their professional expertise and roles as health messengers are currently under-employed in our society-wide response to this crisis. Live-virtual, evidence and competency-based courses, regionally specific courses have the potential to change health professional behaviors toward addressing climate impacts on health by increasing communication, adaptation of practice and engagement in climate and health adaptation and mitigation initiatives.

## Data availability statement

The original contributions presented in the study are included in the article/Supplementary material, further inquiries can be directed to the corresponding author.

## Author contributions

CS, NH, HC, PH, KP, LD, and JH contributed to conception or design of the work, acquisition, analysis, and interpretation of data for the work. All authors contributed to the article and approved the submitted version.
