# Supplementary material for: Climate and health capacity building for health professionals in the Caribbean: A pilot course
Source: Front Public Health. 2023 Jan 26;11:1077306. doi: 10.3389/fpubh.2023.1077306 (PMC9909391; doi:10.3389/fpubh.2023.1077306)
Supplement: Supplementary file 1 [file Table_1.DOCX]

**Appendix 1: Syllabus of *The Caribbean Climate and Health Responder Course***

|  |  |
| --- | --- |
| Main Session | Topics Covered |
| - Session 1: Climate Change for the Health Professional | - Difference between natural climate variability and long-term climate change - The measurement and evidence base of climate drivers - Distinguishing “climate” and “weather,” and between climate change and climate variability - The general mechanism of the greenhouse effect - The social dimensions of climate drivers, including population growth and economic growth - Degree of scientific consensus on climate change. - The main health impacts of climate change, how the health sector contributes to climate change, and what health professionals can do |
| - Session 2: Extreme Weather Hazards: Hurricanes | - The ways in which climate change increases the risk of extreme events such as hurricanes - Short-term and long-term health threats to patients impacted by extreme weather events and steps that health professionals can take to reduce these risks - The roles of disaster risk reduction, public health communication, early warning and regional cooperation in the prevention of the health impacts of extreme weather events - How the health impacts of climate change will vary within and among different communities by applying concepts of vulnerability, resilience and adaptive capacity |
| - Session 3: Water-and Food Related Illness (Flooding & Drought) | - How climate change impacts the hydrosphere and the implications for water availability, quality and supply - How to apply knowledge of climate impacts on the hydrologic cycle to the incidence and prevalence of waterborne pathogens including bacteria, parasites and viruses - Case examples of direct impacts of hydrologic changes on salmonella, norovirus, campylobacter, cryptosporidium, leptospirosis, and vibrio, ciguatera poisoning - How changes to the hydrologic cycle may impact patient health, and steps health professionals can take to reduce this risk - Factors that contribute to individual and community-level vulnerability |
| - Session 4: Temperature Related Illness and Mortality | - Connections among climate, extreme heat, human health and well-being - Climate change, extreme heat and impacts on socio-ecological systems - Hazards, risks and vulnerabilities to extreme heat in a changing climate |
| - Session 5: Degraded Air Quality (Bush Fires & Urban Air Quality) | - Pathways through which climate change affects ozone, PM2.5, and other ambient respiratory irritants and how these pollutants impact climate-sensitive respiratory diseases - How climate change might impact indoor air quality - How climate change makes air quality regulation more complex and difficult - Identify populations that are vulnerable to degraded air quality. How health professionals can protect these vulnerable patients - How wildfires/bushfires are impacted by climate change and the direct and indirect health implications - How climate change increases the risk of complex disasters due to combined and cascading events (heatwaves followed by wildfires) - Identify particularly vulnerable patients and families and teach about risk mitigation, such as limiting outside work and recreation during poor air quality days |
| - Session 6: Vector-borne and Zoonotic Disease | - The environmental processes changing as a result of climate change and how they impact the prevalence, incidence, and distribution of vector-borne and zoonotic diseases. - How climate change is influencing distribution of Leptospirosis, Dengue fever, Chikungunya, Zika, and others - Effects of deforestation/bush fires/climate change bringing forest habitat in closer proximity to humans - What actions health professionals can take to protect patients vulnerable to these diseases - Identify vulnerable populations including: women, outdoor workers, children, immunocompromised - Steps the health sector can take to become prepared to address shifting geographic burdens of vector-borne disease, including increasing surveillance and early-warning systems |
| - Session 7: Mental Health | - How natural disasters resulting from climate change impact the mental health of a population, specifically in regard to the incidence and prevalence of stress disorders, depression, domestic abuse, violence and aggression, and substance abuse - The impact on the mental health of “climate refugees” or those that have been displaced from their home or livelihood from climate related environmental changes such as drought, sea level rise, wildfires or hurricanes - What can be done to mitigate the population mental health consequences of climate change - The unequal burden of climate related mental health disorders and strategies for targeted interventions in vulnerable populations |
| - Session 8: Health System Resilience in a Changing Climate | - How vulnerability assessments are used to understand individual, community and health system impacts and vulnerabilities - How to apply principles outlined in WHO guidance documents to measure the resilience of health systems - How stress testing of health systems facilitates preparedness for climate change - How to use knowledge of vulnerability and adaptive capacity to explore challenges faced by small and remote health systems |
| - Session 9: Healthcare Sector Mitigation | - How to apply the concepts of mitigation and adaptation to the healthcare sector and explore examples of how healthcare systems can perform both - Ways in which health care facilities can become more resilient in the face of increasingly severe and/or frequent climate-related weather extremes. Sensitization to the PAHO SMART Hospital program - How to use emergency planning skills to plan for and respond to climate-related extreme weather events and disasters, including workforce surge needs, and distinguish the roles of and interactions between agencies involved in emergency care - How health professionals can partner with health care institutions, professional organizations, and advocacy groups to reduce healthcare-sector greenhouse gas footprint. |
| - Session 10: Climate Change and Health Equity | - Defining climate-health vulnerability and climate resilience. How to identify social and environmental determinants of health that make individuals and communities more vulnerable to climate-related health threats - Applying the vulnerability framework to specific populations (women, workers, climate refugees, indigenous people) - Describing the unique vulnerabilities of the following populations: elderly, children, socioeconomically disadvantaged, homeless, immunocompromised patients, patients with chronic medical conditions/ NCDs - How to apply knowledge of the ethical, professional, and legal obligations relevant to climate and health |
|  | |
| Skills and Practice Sessions | |
| - Session 1: Greening Your Health Practice | |
| - Session 2: Climate and Health Communication | |
| - Session 3: Clinical Case – Heat Stress | |
| - Session 4: Clinical Case – Flooding | |
| - Session 5: CIMH Session | |

**Appendix 2: Pre- and Post-course Surveys**

| Question | Pre-course Survey | Post-course Survey |
| --- | --- | --- |
| Q1. How often do you talk to your patients/community members/colleagues about climate change and health?   - Frequently - Sometimes - Rarely - Never | Yes | Yes |
| Q2. How often do you incorporate climate change and health knowledge and skills in your work?   - Frequently - Sometimes - Rarely - Never | Yes | Yes |
| Q3. How confident are you that you can engage with a climate and health initiative (e.g. hospital green team, adaptation project, education) in your community/institution/practice?   - Very confident - Somewhat confident - Not very confident - Definitely not confident | Yes | Yes |
| Q4: Do you think that this course has prepared you to speak with patients/community members about climate change and their health?   - Yes, I feel very prepared to have conversations with all contacts - Yes, I feel prepared but in limited scenarios - No, I do not feel like I have the expertise to speak with others on this subject | No | Yes |
| Q5: Do you think that the knowledge and skills you gained from the Climate and Health Responder Course will change your professional practice?   - Yes, it will change my practice to a large degree - Yes, it will change some aspects of my practice - Not sure if it will change my practice - My practice will not change | No | Yes |
| Q6: Do you think the knowledge that you gained from the Climate and Health Responder Course has prepared you to lead climate and health ADAPTATION initiatives within your community of practice?   - Yes, I now feel confident leading initiatives - I feel more confident, but still feel like I need more knowledge/experience to serve as a leader - I do not feel confident to serve as a leader, but am more prepared to help in initiatives - I do not feel confident to lead or help develop initiatives | No | Yes |
| Q7: Do you think the knowledge that you gained from the Climate and Health Responder Course has prepared you to lead climate MITIGATION initiatives within your community of practice?   - Yes, I now feel confident leading initiatives - I feel more confident, but still feel like I need more knowledge/experience to serve as a leader - I do not feel confident to serve as a leader, but am more prepared to help in initiatives - I do not feel confident to lead or help develop initiatives | No | Yes |
| Q8: How confident are you now, as compared to before you took the Climate and Health Responder course, that you can train others in at least some aspects of climate change?   - My confidence has increased a great deal - My confidence has increased slightly - My confidence has not changed - My confidence has decreased slightly - My confidence has decreased a great deal | No | Yes |
